# The Functionalized Single-Walled Carbon Nanotubes Gas Sensor With Pd Nanoparticles for Hydrogen Detection in the High-Voltage Transformers

**DOI:** 10.3389/fchem.2020.00174

**Published:** 2020-04-07

**Authors:** Sirui Tang, Weigen Chen, He Zhang, Zihao Song, Yanqiong Li, Yu Wang

**Affiliations:** ^1^State Key Laboratory of Power Transmission Equipment and System Security and New Technology, Chongqing University, Chongqing, China; ^2^School of Electronic and Electrical Engineering, Chongqing University of Arts and Sciences, Chongqing, China; ^3^Shanghai Urban Construction Vocational College, Shanghai, China

**Keywords:** single-walled carbon nanotubes, Pd-doped, hydrogen detection, gas sensing properties, high-voltage transformers

## Abstract

Single-walled carbon nanotubes (SWCNTs) have been widely discussed and applied as novel gas sensing nanomaterials. Hydrogen is one of the remarkable fault characteristic gases in high-voltage oil-paper insulated transformers. In this paper, 3.07 wt% Pd nanoparticles (NPs) were used to decorate SWCNTs. The unloaded, the carboxylated, and the Pd-doped SWCNTs were fabricated into three planar gas sensors, and their gas sensing properties to hydrogen were studied. Gas sensing mechanism was analyzed. Results show that the optimal operating temperature of a Pd-doped SWCNTs-based gas sensor is 125°C lower than that of the unloaded SWCNTs-based gas sensor, and it shows the highest gas sensing response value. This is attributed to the decreasing work function of Pd, which reduces the hole carries in the nanotubes.

## Introduction

Novel gas sensing materials were discovered and have been studied over the past five decades. A number of works on the improvement of microstructures, structures, and sensing properties of gas sensing materials have been done by researchers. Nanomaterials, such as nanofibers, nanowires, carbon nanotubes (CNTs), and nanoparticles, are the main focus of this research. In a study by Zhou et al. (2017a,b), the highly porous NiO nanodisks (NiO-NDs) and its synthesis, characterization, and sensing applications to alcohol were analyzed. In the reference (Zhou et al., 2018), the 1D hierarchical p-n heterostructured Mn_3_O_4_/SnO_4_ hybrid materials (HMs) was synthesized by Zhou et al., and the sensing results to acetone indicates the perfect gas sensing performance of hybrid materials.

Due to CNTs fullerene structure and its large surface area, and the excellent electrical, mechanical, and thermal properties they have, CNTs have been one of the most widely studied gas sensing materials in the past two decades (Chen et al., 2001; Rana et al., 2017; Zaporotskova et al., 2017). Single-walled carbon nanotubes (SWCNTs) and multi-walled carbon nanotubes (MWCNTs) are the two main types of CNTs. The microstructure of SWCNTs and MWCNTs are somehow the same, that is, they consist of a rolled-up single sheet of a layer of graphene. However, MWCNTs are composed of concentric tubes of graphene fitted inside each other (Pitroda et al., 2016; Beitollahi et al., 2018; Han et al., 2019). It is confirmed that adsorption of electron withdrawing (e.g., NO_2_, O_2_) or donating (like NH_3_) molecules on SWCNTs will cause the charge transfer between the nanotubes and molecules (Kong et al., 2001). Compared with other gas sensing materials, like MOS, CNTs—especially single-walled carbon nanotubes—have remarkable properties. For example, they have the highest Young's modulus, highest thermal conductivity, ballistic electron transport, and a high aspect ratio structure. What's more, CNTs are a more stable electrode material than other gas sensing materials due to its lower probability to be reduced or oxidized during a substantial range of potentials (Robertson, 2004). In the reference (Naje et al., 2016), the detection of NO_2_ using SWCNTs and MWCNTs on porous silicon wafers was done by Naje et al. The NO_2_ gas sensing performance of SWCNTs and MWCNTs vary at temperatures ranging from 25 to 250°C, and it shows that equal sensitivity can be reached with a higher temperature for SWCNTs compared to MWCNTs, while the highest response of SWCNTs (79.8%) is higher than MWCNTs (59.6%) at their optimum temperature (150°C for SWCNTs, 200°C for MWCNTs). The trace level detection of NH_3_ and NO_2_ at room temperature via randomly oriented SWCNTs, which is grown by PECVE technique at 650°C, were realized by Lone et al. (2018). Results show the quick response and recovery characteristics of both NH_3_ and NO_2_. Additionally, the gas sensing abilities of unloaded SWCNTs to N_2_O_4_ (Dai et al., 1999), O_2_ (Kong et al., 1998), CO_2_ (Yoon et al., 2018), and CH_4_ (Poonia et al., 2015) are widely discussed.

High-voltage oil-paper insulated transformers play an essential role in power transmission, but during the long-term operation, due to oxidation, pollution, and excessive inner temperature, the transformer insulation oil will be degraded and decomposed, producing traces of characteristic gases dissolving in the oil. Hydrogen is one of the main characteristic gases reflecting overheat fault and discharge fault in the oil-immersed transformer (International Standard IEC 60599: 2015, 2015). Gas sensing nanomaterials such as SnO_2_, ZnO, WO_3_, and MoS_2_, as well as their functionalized derivatives, have been applied in studies on characteristic gases detecting of high-voltage transformers (Tang et al., 2017; Zhou et al., 2018; Wang et al., 2019; Wei et al., 2019). More importantly, Zhang et al. found that CNTs are well-performed gas sensing materials for gas detection of high-voltage electrical equipment (Zhang et al., 2017). However, it was reported in the study Kong et al. (2001) that nanotubes show a poor sensing response to some gas molecules. Instead, geometrical optimizations of SWCNTs with and without doped metals and their gas adsorption structures were studied based on computational methodology by applying restricted density functional theory (DFT) by Tabtimsai et al. (2011). Calculation and simulation indicated that decorating SWCNTs with Pd nanoparticles can effectively enhance the gas sensing performance to NO_2_, NH_3_, H_2_O, and H_2_. The hydrogen atoms dissociated from the hydrogen molecules have smaller adsorption energy and dissociation energy on the Pd cluster. As an electron donor, Pd clusters can quickly dissociate hydrogen atoms, electron acceptors, and accelerate electron transfer in gas-sensitive materials.

In this paper, the carboxylated and 3.07 wt% Pd-doped SWCNTs-based nanomaterials were synthesized based on the unloaded SWCNTs-based nanomaterials. Three SWCNTs-based nanomaterials (the unloaded, the carboxylated, and the Pd-doped) were fabricated into corresponding planar gas sensors. Gas sensing properties including the temperature characteristics, the concentration characteristics, the linearity, detecting limitation, and the response and recovery time characteristics of three SWCNTs-based gas sensors to hydrogen were studied. Gas sensing mechanisms were analyzed. In the results, Pd-doped SWCNTs-base gas sensor presents the best gas sensing performance to hydrogen. This study can provide a novel solution to the issue of characteristic gases detection in high-voltage oil-paper insulated transformers.

## Materials and Methods

### Preparation of SWCNTs-Based Nanomaterials

The unloaded SWCNTs-based nanomaterials were made by Timesnano of Chengdu Organic Chemicals Co. Ltd., Chinese Academy of Sciences. The outer Diameter (OD) of the unloaded SWCNTs-based gas sensing nanomaterials is 1–3 nm, and the purity is all higher than 90 wt%. The length of nanomaterials is about 50 microns. Concentrated hydrochloric acid, concentrated sulfuric acid, concentrated nitric acid, isopropanol, and ammonia used in the preparation process were purchased from Chongqing Chuandong Chemical Co., Ltd. (China), and all were of analytical grade. Palladium chloride (PdCl_2_) provided a source of palladium. Deionized water was also used.

The processes of preparation of SWCNTs-based nanomaterials are classified into three steps:

(1) Purification of the unloaded SWCNTs

Six hundred milligram of unloaded SWCNTs were added to 300 ml of concentrated hydrochloric acid and ultrasonically cleaned for 30 min. Five hundred milligram of deionized water was used to ultrasonically clean the mixture for another 30 min. The cleaned mixture was placed in a drying box and dried at 150°C for 10 h. Purified unloaded SWCNTs were obtained.

(2) Acidification of the purified SWCNTs

Four hundred milligram of purified unloaded SWCNTs were added to 50 ml of concentrated sulfuric acid and 20 ml of concentrated nitric acid, and heated and stirred at 80°C for 4 h. After the mixture was cooled down, it was diluted with 60 ml deionized water. The mixture was filtered through a microporous membrane with a pore diameter of 0.45 microns, and washed repeatedly with deionized water until pH = 7. After drying at 80°C for 12 h, acidified SWCNTs were obtained.

(3) Functionalization of the acidified SWCNTs

Two hundred milligram of SWCNT was dissolved in 50 ml of isopropanol and sonicated for 20 min to improve the dispersibility of SWCNTs. 10.31 mg of PdCl_2_ was dissolved in 3 ml of ammonia water. PdCl_2_ solution was added to the SWCNTs/isopropanol mixed solution drop by drop, and stirred at a high speed for 2 h. The obtained suspension was put in a drying box and dried at 80°C for 2 h. The obtained powder was put in a calciner at 600°C for 2 h. 3.07 wt% Pd-doped SWCNTs-based nanomaterials were synthesized successfully.

The microstructure of the nanomaterials was characterized by Scanning Electron Microscopy (SEM) (SU8020, HITACHI, Japan) and Transmission Electron Microscopy (TEM) (JEM-2000EX, JEOL, Japan).

### Fabricated of SWCNTs-Based Gas Sensors

**Figure 1:**
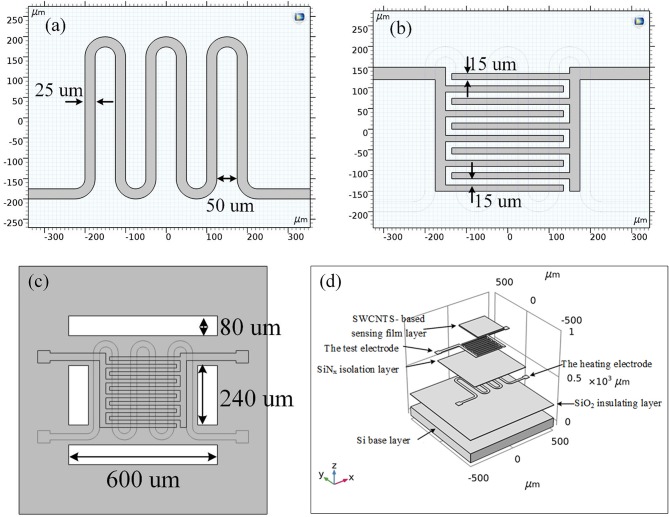
**(a–c)** The 2D structure of the planar gas sensor. **(d)** The 3D structure of the planar gas sensor.

**Figure 2:**
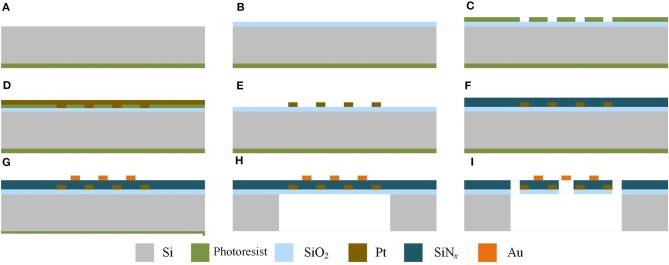
Steps of processing of the planar gas sensor in sectional view. **(A)** The productive layer of Si base layer productive. **(B)** SiO_2_ insulting layer is produced by thermal oxidation. **(C)** Photolithographing on the heating electrode. **(D)** Sputter the Pt electrode. **(E)** Remove the metal-attached photoresist by stripping. **(F)** Deposition of SiN_x_ isolation layer on the heating electrode. **(G)** Photolithography, sputtering and stripping of Au electrode. **(H)** Corrosion of Si base layer. **(I)** Etching of SiO_2_ insulating layer and SiN_x_ isolation layer.

Planar gas sensors are applied in this paper. The structure of planar gas sensors is shown in Figure 1. The planar gas sensor is mainly composed of a Pt heating electrode, a Si base layer, an Au gas sensing electrode, and a SWCNTS-based sensing film layer. The size of the testing area is 300 μm ×300 μm. The hardware structure of the gas sensor is fabricated by Zhengzhou Winsen Electronics Technology CO., Ltd by processes mainly including oxidation, photolithography, sputtering, stripping, deposition, and etching, which are presented in Figure 2.

**Figure 3:**
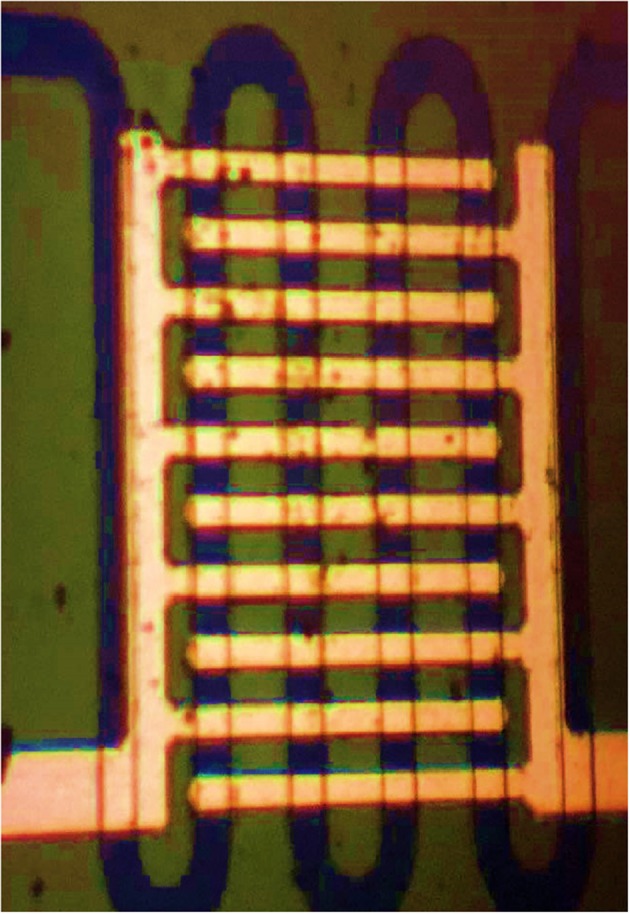
The planar gas sensor.

The planar sensor array is shown in Figure 3. The n-type (110) crystal face double polished silicon wafer (thickness is about 300 μm, and conductivity is 0.001–0.1 S/m) is used. In this paper, three SWCNTs-based sensing nanomaterials are coated by the droplet guiding method. After fully grinding the appropriate number of SWCNTs-based nanomaterials, it was dissolved in absolute ethanol for 1 h to obtain the corresponding dispersion, which was then dried at 400°C to get ultrafine powder. The deionized water droplets were applied to the sensor unit testing area using a micro syringe, and the SWCNTs-based nanomaterial powder was carefully applied to the droplets. After drying for 8 h, the powder was closely attached to the testing area, and three SWCNTs-based planar gas sensors were fabricated successfully.

### Gas Sensing Test Methods

**Figure 4:**
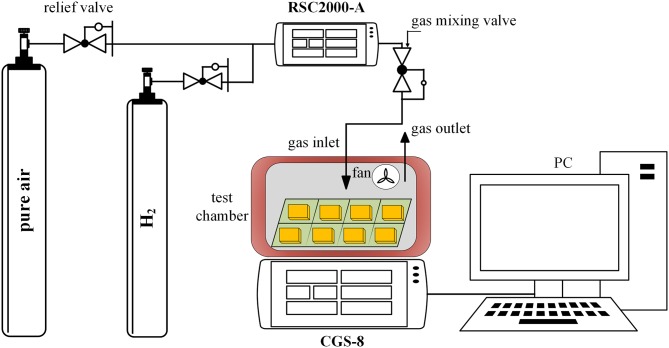
The experimental platform.

**Figure 5:**
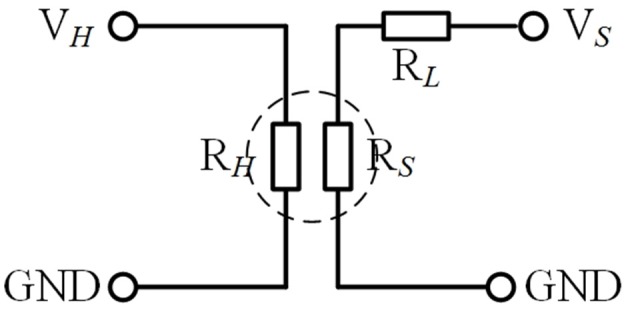
The test circuit.

The experimental platform consists of air source, RSC2000-A automatic gas mixing system (Beijing JS Co.), and CGS-8 intelligent gas sensitivity analysis system (Beijing Elite Tech Co., China), which is presented in Figure 4. The detection of the electrical signals of the gas sensor is mainly realized by the classical resistor divider principle, and the test circuit is shown in Figure 5, where *R*_*L*_ is the adjustable load resistance and *R*_*S*_ is the resistance at both ends of the test electrode. *R*_*H*_ is the resistance of the heating electrode of the sensing unit. *V*_*H*_ and *V*_*S*_ is the heating voltage and test voltage, respectively. During the test, *R*_*S*_ will vary with the change of condition and can be calculated by the principle of voltage division.

The sensor response values defined here are as follows (Wang et al., 2010):

*R*_*s*_ = (*R*_*gas*_−*R*_*air*_)/*R*_*air*_

Specific steps are as follows:

1. The gas sensor is placed in a closed air chamber filled with pure air (20 L), and the sensing signal (*R*_*s*_) is recorded by CGS-8 system during the test.
2. The determined concentrations of hydrogen are injected by RSC2000-A system (gas flow: 250 sccm). The resistance value is recorded after the signal is stable.
3. The air chamber is opened through the fume hood and the resistance made to return to the initial value *R*_*air*_.
4. Step (2) and step (3) are repeated.
5. The recorded data is saved and further experiments can be proceeded with.

Experiments were all carried out at a temperature of 28°C and at 60% humidity.

## Results and Discussions

### Morphology

**Figure 6:**
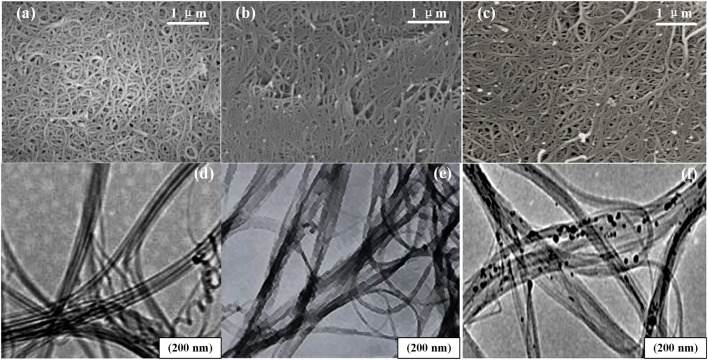
The morphology of nanomaterials. **(a–c)** The SEM images of the unloaded, the carboxylated, and the Pd-doped SWCNTs. **(d–f)** The TEM images of the unloaded, the carboxylated, and the Pd-doped SWCNTs.

Figure 6 shows the SEM and TEM images of the unloaded, the carboxylated, and the Pd-doped SWCNTs. From the SEM images, the three kinds of SWCNTs are all intertwined and woven into a mesh, and the morphology of the SWCNTs after functioning has not changed much. However, in Figures 6b,c, the carboxylated and the Pd-doped SWCNTs are shorter in length and more distributed compared to the unloaded SWCNTs in Figure 6a. In the TEM images, all three SWCNTs are curved and tubular, and the basic structure has not been functionally damaged.

After acidification treatment, SWCNTs were oxidized under the action of concentrated sulfuric acid and concentrated nitric acid. Oxygen atoms released by concentrated nitric acid attacked carbon nanotubes, especially the defects at the ends and on the tube walls. Because carbon atoms are not stable six-membered rings in SWCNTs and are in a metastable state, SWCNTs will break in places where the nanotube curvature is large. Thus, the length of nanotubes becomes shorter, and nanotubes gradually disperse.

### Gas Sensing Properties

The temperature characteristics, concentration characteristics (including linearity and detecting limitation), response and recovery time characteristics of the unloaded, the carboxylated, and the Pd-doped SWCNTs- based gas sensors were studied in the hydrogen atmosphere.

**Figure 7:**
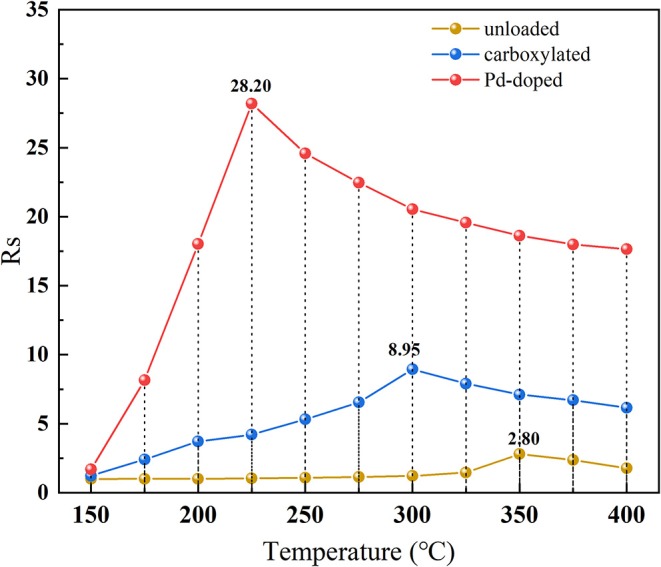
The temperature characteristics of the unloaded, carboxylated, and 3.07 wt% Pd-doped SWCNTs-based gas sensors.

In Figure 7, the temperature characteristics of three SWCNTs-based gas sensors are illustrated among 150–400°C to 100 μL/L hdrogen. On the one hand, three different SWCNTs-based gas sensors present the same trend of temperature characteristics: curves climb at first with the increase of the temperature and decrease after reaching the optimal operating temperature. This might be because when heated to a certain temperature, the surface of SWCNTs nanomaterials is occupied by oxygen atoms released from the air, which increases the surface conductance of the materials and, thus, shows a decrease in resistance. On the other hand, after modifying SWCNTs-based nanomaterials with Pd nanoparticles, the optimal operating temperature dropped sharply by 125°C, and the peak response of Pd-doped SWCNTs-based gas sensor is 10.1 and 3.2 times higher than that of the unloaded and carboxylated SWCNTs-based gas sensors under the respective optimal operating temperature, respectively.

**Figure 8:**
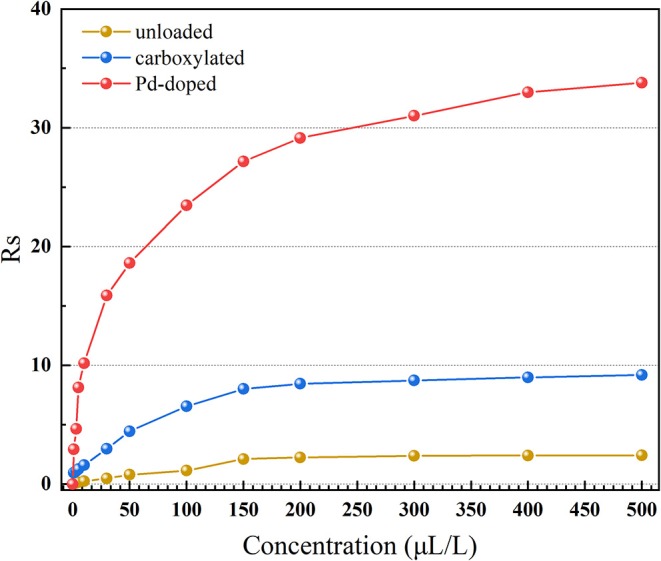
The concentration characteristics of the unloaded, carboxylated, and 3.07 wt% Pd-doped SWCNTs-based gas sensors.

Concentration characteristics were tested in 0–500 μL/L hydrogen at 275°C, as shown in Figure 8. As the gas concentration rises, the growth rate of the response value of all three gas sensors slows down and gradually becomes saturated. However, 3.07 wt% Pd-doped SWCNT-based gas sensor has better gas sensing performance in an extreme high gas concentration atmosphere. When the concentration of hydrogen is 500 μL/L, the gas sensing response of Pd-doped SWCNTs-based gas sensor is about 33.79, which is almost 14 and 3.7 times higher than the gas sensing response of the unloaded and the carboxylated SWCNTs-based gas sensors, respectively.

In Figure 8, *R*_*s*_ is linearly related to the concentration in the respective low concentration ranges (1–30 μL/L), and the fitting function and corresponding *R*^2^(linearity) is shown in Table 1. Assuming that when hydrogen is 1 μL/L, the gas sensing response is not lower than 1, the corresponding gas sensor is able to reach the detecting limitation (International Standard IEC 60599: 2015, 2015; Tang et al., 2017). Results are shown in Table 1. SWCNTs-based gas sensors with or without functionalization all show perfect linearity, while only the Pd-doped SWCNTs-based gas sensor can meet the requirement of detecting limitation.

**Table 1:** The fitting function, linearity and detecting limitation of three SWCNTs-based gas sensors to hydrogen.

**Sensor units**	**The fitting function**	**R^2^**	**Detection limit**
Unloaded	*y* = 0.0148*x* +0.0574	0.994	**×**
Carboxylated	*y* = 0.0706*x* + 0.8921	0.9999	**×**
Pd-doped	*y* = 1.2757*x* +0.9729	0.9697	✓

**Figure 9:**
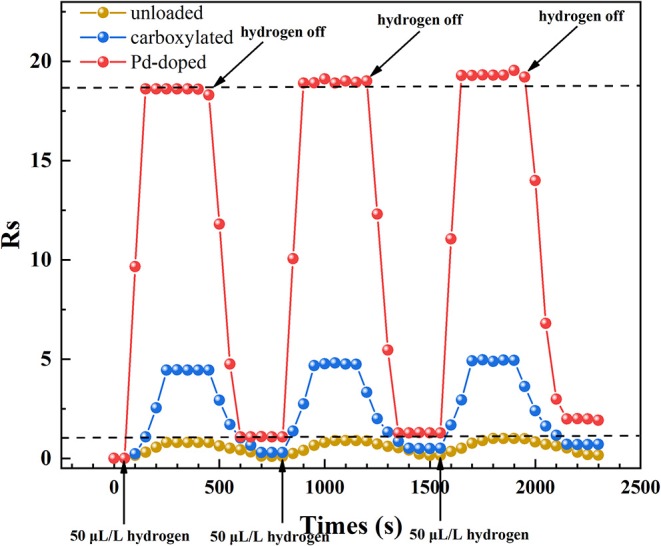
The gas response and recovery time characteristics of three different SWCNTs-based gas sensors.

Figure 9 presents the response and recovery time characteristics of three gas sensors which were tested at 275°C to 50 μL/L hydrogen. The experiment was repeated three times: 50 μL/L hydrogen was injected at 50, 800, and 1,550 s, and was exhausted at 450, 1,200, and 1,950 s. It is obvious that both response and recovery time of Pd-doped SWCNTs-based gas sensor are shorter than the other two. The response time and recovery time of Pd-doped SWCNTs-based gas sensor are about 100 and 150 s. What's more, the gas sensing response value of the three gas sensors is slightly increased after the third time test, which might be because of the incomplete exhaust each time, resulting in the presence of more hydrogen than expected in the final gas chamber. The recovery process of gas sensing materials is a dynamic process of adsorption and desorption between the molecules of the test gas, hydrogen, and nanotubes. If the hydrogen molecules in the gas chamber are not exhausted cleanly, a small amount of hydrogen molecules will still be adsorbed with the active sites on the surface of the nanotube at high temperatures, shown as the hydrogen atoms, so that there will still be less electron exchange in the gas sensing material nanotubes.

### Gas Sensing Mechanism

Obviously, after decorating SWCNTs with Pd nanoparticles, the gas sensing performance to hydrogen was remarkably enhanced. This is attributed to the enhanced electrical property toward molecular hydrogen compared with the undecorated SWCNTs. The gas sensing reaction is the interactions between H_2_, Pd, and the nanotubes. After heating, hydrogen molecules dissociate faster into atomic hydrogen on Pd surface, resulting in the dissolution of atomic hydrogen in Pd with high solubility, consequently decreasing the work function of Pd, that is shown in the formula (1). The lowering of the work function of Pd leads to faster and easier electron transfer from Pd to SWCNTs, reducing the number of hole-carries in the p-type nanotubes and the value of conductance.

When the Pd-doped SWCNTs-based gas sensor was in a low hydrogen atmosphere, the reason why it can reverse and auto recover is because of the oxygen in the air and on the surfaces, which is represented as the formulas (2) and (3) (Mandelis et al., 1993; Collins, 2000; Kong, 2000).

(1) $$\begin{array}{lll} H_{2}\to 2H_{atom\;on\;surfaces}H_{atom\;on\;surfaces} & \to & H_{atom\;in\;Pd} \end{array}$$

(2) $$\begin{array}{lll} O_{2}+2H_{atom\;on\;surfaces} & \to & 2OH \end{array}$$

(3) $$\begin{array}{lll} {OH+H}_{atom\;on\;surfaces} & \to & H_{2}O \end{array}$$

## Conclusion

In this paper, we prepared the carboxylated and 3.07 wt% Pd-doped SWCNTs-based nanomaterials based on the unloaded SWCNTs, and three different SWCNTs-based planar gas sensors were fabricated and tested in hydrogen to study their gas sensing properties. Results show that functionalized SWCNTs-based gas sensor with Pd nanoparticles present the best gas sensing performance, and has the lowest optimal operating temperature (225°C) and the highest gas sensing response to 500 μL/L hydrogen at 275°C (*R*_*s*_ ≅ 33.79). The gas response and recovery time of Pd-doped SWCNTs-based gas sensor are both 50 s shorter than those of the unloaded and carboxylated SWCNTs-based gas sensors. This was because the Pd doping lowers the work function and enhances the electrical property toward molecular hydrogen. Results could assist the development of novel SWCNTs-based gas sensors for fault characteristic gases detection in the high-voltage electrical transformers.

## Data Availability Statement

The datasets generated for this study are available on request to the corresponding author.

## Author Contributions

ST designed the experiment, finished the experiment, data collected and analyzed, and wrote the paper. WC helped correct the paper. HZ helped design and finished the experiment. ZS helped collect the data. YL helped correct the paper. YW helped correct the paper.

### Conflict of Interest

The authors declare that the research was conducted in the absence of any commercial or financial relationships that could be construed as a potential conflict of interest.
